# Contrasting resistance and resilience to light variation of the coupled oxic and anoxic components of an experimental microbial ecosystem

**DOI:** 10.1002/ece3.8793

**Published:** 2022-04-06

**Authors:** Marcel Suleiman, Frank Pennekamp, Yves Choffat, Owen L. Petchey

**Affiliations:** ^1^ Department of Evolutionary Biology and Environmental Studies University of Zurich Zürich Switzerland

**Keywords:** light disturbance, microbial communties, oxygen dynamics, resilience

## Abstract

Understanding how microbial communities of aquatic ecosystems respond to environmental change remains a critical challenge in microbial ecology. In this study, we used light‐dependent oxic–anoxic micro‐ecosystems to understand how the functioning and diversity of aerobic and anaerobic lake analog communities are affected by a pulse light deprivation. Continuous measurements of oxygen concentration were made and a time series of full‐length 16S rRNA sequencing was used to quantify changes in alpha‐ and beta diversity. In the upper oxic layer, oxygen concentration decreased significantly under light reduction, but showed resilience in daily mean, minimum, and maximum after light conditions were restored to control level. Only the amplitude of diurnal fluctuations in oxygen concentrations did not recover fully, and instead tended to remain lower in treated ecosystems. Alpha diversity of the upper oxic layer communities showed a delayed increase after light conditions were restored, and was not resilient in the longer term. In contrast, alpha diversity of the anoxic lower layer communities increased during the light reduction, but was resilient in the longer term. Community composition changed significantly during light reduction, and showed resilience in the oxic layer and lack of resilience in the anoxic layer. Alpha diversity and the amplitude of daily oxygen fluctuations within and among treatments were strongly correlated, suggesting that higher diversity could lead to less variable oxygen concentrations, or vice versa. Our experiment showed that light deprivation induces multifaceted responses of community function (oxygen respiration) and structure, hence focusing on a single stability component could potentially be misleading.

## Graphical abstract

In this study, we used light‐dependent oxic–anoxic micro‐ecosystems to understand how the functioning and diversity of aerobic and anaerobic lake analog communities is affected by a pulse light deprivation. Continuous measurements of oxygen concentration were made and time series of full‐length 16S rRNA sequencing were used to quantify changes in alpha‐ and beta diversity. Our experiment showed that light deprivation induces multifaceted responses of community function and structure, hence focusing on a single stability component could potentially be misleading.

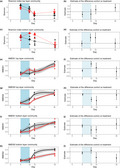

## 1 INTRODUCTION

Microbial communities are critical components of ecosystems, driving their development and functioning for billions of years. Over these long timescales, microbes have faced major transitions, such as the increasing oxygenation of Earth's atmosphere. The success of microbial life is to a large degree explained by being highly adaptable to a variety of environmental conditions (Allison et al., 2013; Evans & Wallenstein, 2014). Even slight changes in conditions can lead to taxonomical and functional community shifts, which themselves provide indications of microbial community stability, or lack thereof (Shade et al., 2012). Communities can react toward pulse disturbances by showing resistance, resilience, functional redundancy, and alternate stable states (Allison & Martiny, 2009). Observing responses on the functional and compositional level and how they are related can provide much needed insights about the mechanisms of microbial community stability (Philippot et al., 2021). How functioning and stability are mediated by the diversity of an ecosystem has been addressed with controlled experiments (Fox, 2013; Huston, 2014; Yachi & Loreau, 1999), however, these assume random species loss, while disturbances often affect biodiversity nonrandomly (De Laender et al., 2016).

Lake ecosystems are facing a broad range of stressors (Chaudhari et al., 2018), including temperature increase, fertilizer intake (Gao et al., 2015), chemical pollutants (Li et al., 2019), or increasing microplastic pollution (Andersson & Anderson, 1980). One factor that is still not investigated well is light reduction in lake ecosystems (Piwosz et al., 2020), which can occur indirectly due to biomass formation on aquatic surfaces, including blooms of algae (Sun et al., 2008) and biofilms (Jones et al., 2002), browning (Scharnweber et al., 2021), snow cover (Garcia et al., 2019) as well as plant biomass growth. A broad range of functional aerobic and anerobic microbial groups are highly dependent on light, including cyanobacteria in the upper water column and phototrophic bacteria in the lower water column. Hence, light reduction could have a large effect on microbial community composition and ecosystem properties such as the oxygen concentration in lake ecosystems (Bush et al., 2017) due to tight coupling of light, microbial respiration, and cyanobacterial O_2_ production.

Here, we studied the response of mixed aerobic–anaerobic ecosystems to a pulse reduction in light intensity using a recently developed dynamic phototrophic oxic–anoxic micro‐ecosystem (Suleiman et al., 2021). These micro‐ecosystems are analogs of freshwater ecosystems, like lakes and ponds, which harbor highly diverse functional groups of microorganisms, tightly connected through the oxygen state of the aquatic environment, which are dependent on light for oxygenic and anoxygenic photosynthesis.

## 2 MATERIAL AND METHODS

Sediment and water samples were taken from a pond (Zurich, Switzerland, 47°23′51.2″N 8°32′33.3″E) and eight micro‐ecosystems (diameter 13 mm, height 16 cm) were set‐up as reported previously (Suleiman et al., 2021; Figure A1). In short, 1.5 cm of sediment (with 0.5% crystalline cellulose, 0.5% methyl‐cellulose, 1% CaSO_4_, 0.2% CaCO_3_, 0.01% NH_4_H_2_PO_4_) was covered with 16 ml pond water and incubated for 35 days at 24°C under a light–dark cycle of 16:8 h (gradient of light). Continuous noninvasive oxygen measurements (PreSens Precision Sensing GmbH, Germany) were performed every 5 min at the lower liquid part (1.5 cm above sediment) and upper liquid part (2 cm below surface) of each column. After incubating the eight columns for 8 days, four columns were covered tightly with aluminum foil and incubated in darkness for 7 days (stressor treatment). After the treatment, incubation continued with the standard light conditions (light–dark cycle of 16:8 h), like the control group, for another 20 days. Five hundred microlitre of liquid sample was taken on day 8 (prior to stressor), day 15 (stressor sample), day 19 (short‐term recovery), and day 35 (long‐term recovery), respectively, at the height of the top and bottom oxygen sensor. DNA extraction and 16S rRNA full‐length sequencing (PacBio) were performed as reported previously (Suleiman et al., 2021), using the primer pair 27F (5′‐AGRGTTYGATYMTGGCTCAG‐3′) and 1592R (5′‐RGYTACCTTGTTACGACTT‐3′). Raw sequencing data were transcribed to Amplicon Sequence Variants (ASV) with *Dada2* (Callahan et al., 2016), and analysis of alpha‐ and beta diversity were performed with the R packages *phyloseq* (McMurdie & Phyloseq, 2013) and *vegan* (Oksanen et al., 2019).

In order to compare the effects of the light treatment, we calculated and analyzed seven response variables (daily mean, maximum, minimum, and amplitude of oxygen concentration, alpha diversity (Shannon index), and two components of microbial community composition). Microbial community composition was quantified using NMDS (nonmetric multidimensional scaling) based on Bray–Curtis distances with the *metaMDS* function of the vegan R package (Oksanen et al., 2019), with two dimensions used (giving the previously mentioned two components of microbial community composition).

Each of the seven response variables was analyzed separately at each time point it was measured at. At each time point, we calculated the mean of each treatment group (control group and light reduction group), the difference between these means, and the 95% confidence interval of this difference (assuming normally distributed errors). We then visually inspected if the confidence interval included zero, and if so judged there to be no difference between the treatment and control group, and if so that there was a difference. We say that a response variable was resistant to the treatment if the 95% confidence interval of the difference between control and treatment included zero at the end of the treatment (day 15 and 19) (or not resistant if the 95% CI did not include zero). We say that a response variable was resilient to the treatment if the 95% confidence interval of the difference between control and treatment included zero at the end of the experiment (day 35; or not resilient if the 95% CI did not include zero). Although the oxygen measurements are plotted and analyzed for each day, we only based assessments of resistance and resilience on the same days as for community composition (days 15, 19, 35). This reduces the number of comparisons, which reduces any issues associated with multiple testing, and also reduces any issues associated with temporal autocorrelation. Detailed scripts are available on zenodo (10.5281/zenodo.5195092) and sequencing raw reads are available on NCBI (PRJNA731625).

## 3 RESULTS AND DISCUSSION

**FIGURE 1:**
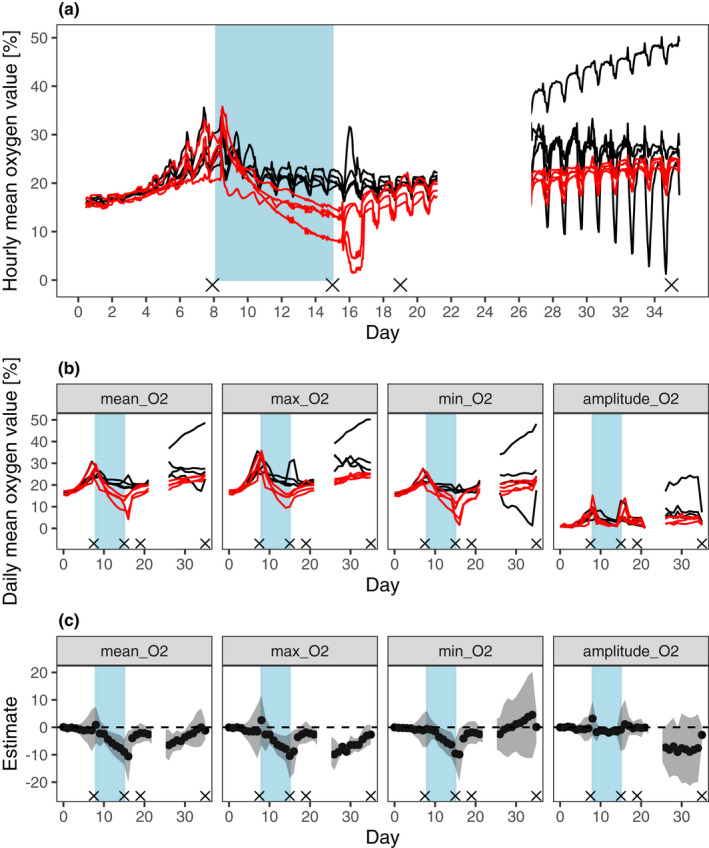
Dynamics and response of oxygen in the micro‐ecosystems’ top layers. Oxygen concentration was recorded every 5 min. (a) Hourly mean of the oxygen concentration of the top sensors of the eight micro‐ecosystems. (b) Daily mean oxygen concentration (illustrated as mean, maximum, minimum, and amplitudes) of the top sensors of the eight micro‐ecosystems. Black lines represent controls, red lines represent columns incubated in darkness from days 8 to 15 (blue area). (c) Estimates of difference in the mean and 95% CI of the estimate of the oxygen concentration treatment versus controls. Gray ribbons show the 95% confidence intervals. Crosses show the sampling days for analyzing microbial communities of the upper and lower layers. A recording error caused the missing oxygen data from days 23 to 27

**FIGURE 2:**
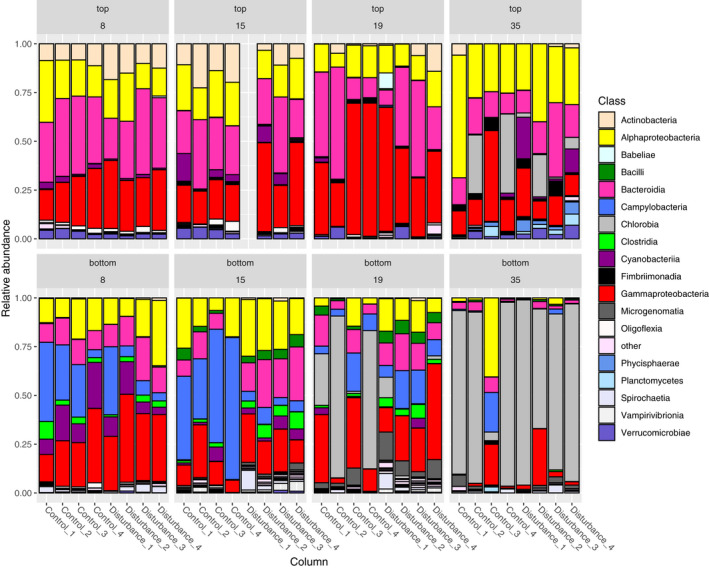
Microbial community composition of the micro‐ecosystems. Relative abundance of microbial community composition (rel. abundance >5%) at class level for the upper (top) and lower (bottom) layer communities of the micro‐ecosystems. Controls were incubated under a light–dark cycle of 16:8 h for 35 days. Disturbed columns were incubated under light–dark cycle for 8 days (prior stressor sample), then incubated in darkness until day 15 (stressor sample), before incubating again under a light–dark cycle of 16:8 h (short‐term recovery sample on day 19 and long‐term recovery sample on day 35)

All eight micro‐ecosystems showed oxygen‐based stratification, resulting in an oxic top layer (Figure 1a) and anoxic bottom layer (Figure A2). The detected microbial communities and their compositions were characteristic for layered lake ecosystems, with aerobic communities in the upper oxic layer (diverse members of *Gammaproteobacteria*, *Bacteroidia*, *Cyanobacteria*) and (phototrophic) anoxygenic phototrophs in the anoxic lower layer (*Chlorobium*, *Chlorobaculum*, *Magnetospirillum*, *Sulfuricurvum*; Figures 2 and A1a).

During the first week, the oxygen concentration of the upper layer of the eight micro‐ecosystems increased during the light phase and decreased during the dark phase, resulting in comparable behavior of the mean‐, max‐, and min‐oxygen concentration, as well as the amplitude (Figure 1, days 0–8). During light reduction (days 8–15) the disturbed columns showed a significant decrease in total oxygen concentration to microaerophilic conditions (Figure 1b, day 8–15). After normal light conditions were restored, oxygen concentration increased, showing resilience until the end of the experiment for mean, minimum, and maximum, but not for the amplitude, which was higher in the controls (Figure 1c). Therefore, light reduction had a lasting impact on the oxygen amplitude, which stayed within a narrower range of values for communities that experienced the stressor, while untreated ones were more variable. A single replicate showed a higher oxygen concentration at the end of the experiment. Exclusion of that microcosm affected the estimated differences in oxygen concentration (i.e., resistance and resilience), but did not qualitatively change the results. Exclusion also had no effect on the conclusions of the analyses of microbial community composition. Since the lower layer turned completely anoxic within 2 days, we did not detect any effect of light reduction on the oxygen pattern there.

**FIGURE 3:**
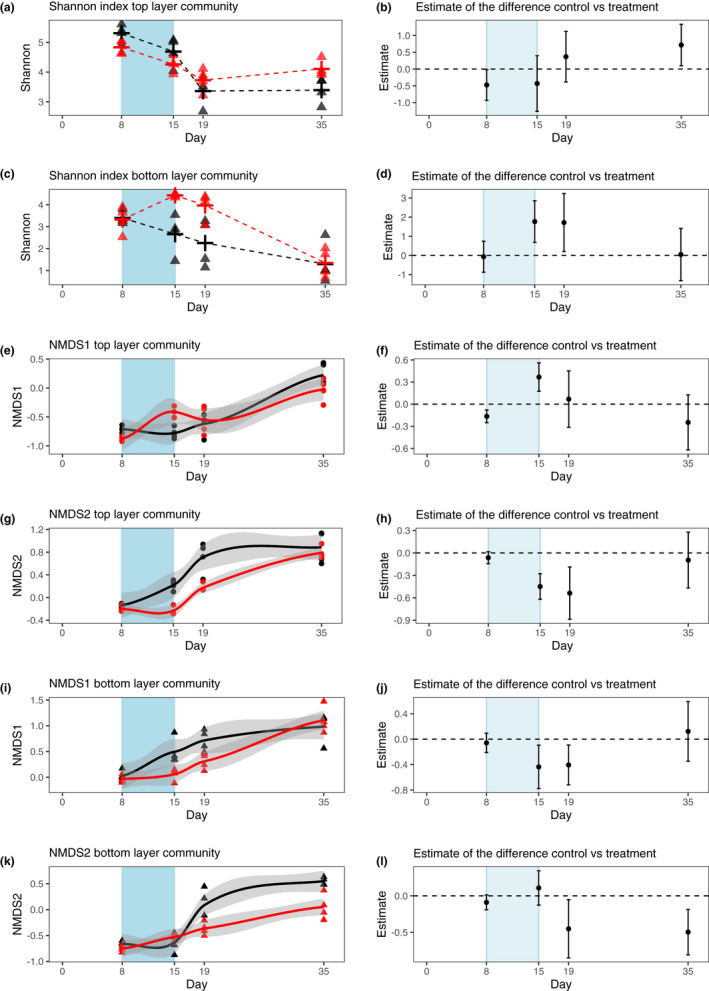
Resistance and resilience of the microbial communities based on full‐length 16S RNA sequencing. (a) Alpha diversity based on Shannon index of the upper water communities of the eight micro‐ecosystems. Cross: mean. (b) Estimated difference between treatments and 95% confidence interval for the alpha diversity analysis for the upper water communities. (c) Alpha diversity based on Shannon index of the lower water communities of the eight micro‐ecosystems. Cross: mean. (d) Estimated difference between treatments and 95% confidence interval for the alpha diversity analysis for the lower water communities. (e, g, i, k) Beta diversity analysis based on NMDS1 and NMDS2 score for the upper and lower water communities, respectively. (f, h, j, l) Estimated difference between treatments and 95% confidence interval for the beta diversity (based on NMDS1 and NMDS2 scores) as response parameters for the upper and lower water communities. Black lines represent controls, red lines represent columns incubated in darkness from days 8 to 15 (blue area). Gray ribbons show the 95% confidence intervals

Alpha diversity was differentially affected by light reduction in the upper and lower layers (Figure 3): The richness of the anaerobic lower communities was immediately increased by light reduction (Day 15, Figure 3c+d) and stayed significantly different after normal light conditions were restored in the short‐term recovery sample, but showed resilience (i.e., return to control values) in the long term. In contrast, the alpha diversity of the aerobic upper layer communities was not immediately affected by the stressor, showed marginally significant differences in the short‐term recovery samples but stayed significantly changed in the long term (Figure 3a+b).

Nonmetric multidimensional scaling revealed significantly different compositions of upper and lower layer communities within a single micro‐ecosystem, but the upper and lower composition converged during light reduction (Figure A3). This convergence is most likely due to the loss of the light‐ and oxygen gradient during the disturbance, indicating how stressors can directly influence microbial communities by affecting stratification processes in an ecosystem.

To understand the changes in community composition, we analyzed time series of NMDS1 and NMDS2 (Figure 3e–l). For the lower community, the NMDS1 score was immediately affected by light reduction but showed resilience, while NMDS2 was affected in delayed fashion and remained significantly changed (Figure 3i–l). The upper layer communities were affected immediately based on NMDS1 and NMDS2, but both showed resilience at the last sampling (Figure 3e–h).

**FIGURE 4:**
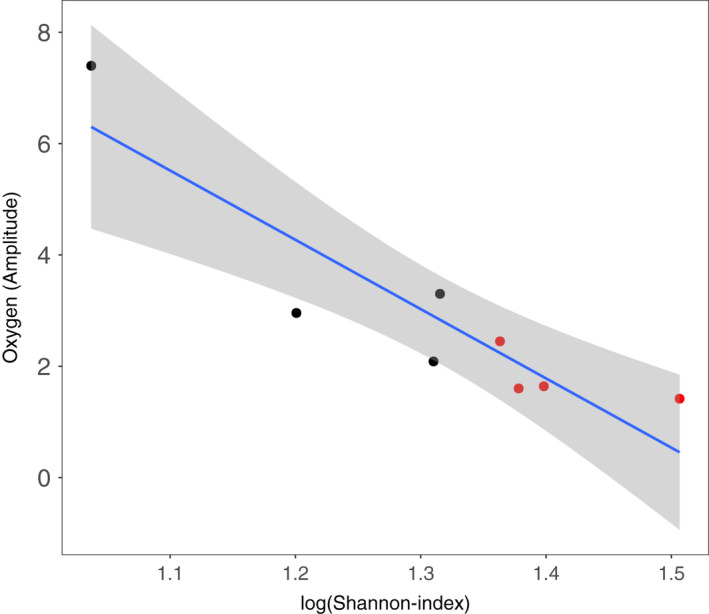
Relationship between the amplitude of daily fluctuations in oxygen concentration and Shannon diversity of upper layer communities the long‐term recovery sample (day 35). Black symbols represent controls, red symbols represent treated columns. Gray ribbons show the 95% confidence intervals. Pearson's product‐moment correlation: *p*‐value = .002, correlation coefficients = −0.90

In order to understand possible relationships between diversity and stability, we examined the correlation of alpha diversity and the oxygen amplitude of the last incubation on day 35 (Figure 4). We found a strong negative correlation between alpha diversity and amplitude of diurnal oxygen concentration (*n* = 8, *t* = −5.1134, *p* = .002). This correlation holds when excluding the replicate with high mean oxygen concentration, very high amplitude of oxygen variation (~7.4), and low diversity (SI 2.8; *n* = 7, *t* = −2.6, *p* = .04; Figure A4). This finding is in line with the insurance hypothesis of biodiversity, that higher biodiversity will cause lower temporal variation in aggregate properties of communities and of ecosystem states such as oxygen concentration (Yachi & Loreau, 1999). However, in our experiment diversity was not manipulated, but was rather an outcome of the factors such as environmental conditions (including the light treatment) and interspecific interactions. Accordingly, we cannot be sure if the higher diversity observed in the light reduction treatment was the cause of the greater stability (lower amplitude) of the oxygen concentration in these communities. Also, since, we did not manipulate the amplitude of the oxygen fluctuations, we cannot say if this was responsible for the observed differences in alpha diversity. Further studies manipulating the diversity of organisms in the ecosystem and separately the magnitude of fluctuations in environmental conditions such as oxygen concentration would be needed to assign causation and understand the likely feedback between organismal diversity and environmental fluctuations.

Our experiment showed that light reduction induces multifaceted responses of community function and structure at multiple temporal scales. We found that light reduction has differential effects on the coupled communities in ecosystems: (i) light reduction had a strong effect on microbial function (oxygen consumption/production), richness and composition, but most responses were resilient in the long run (ii); For nonresilient aspects, we found that the richness and the amplitude of oxygen concentration were related. We suggest that nonrandom effects of light reduction leads to a change in richness, which subsequently affects the oxygen amplitude, but this proposed causal relationship needs experimental testing (iii); recovery and resilience operate at different timescales and hence several time points of analysis are needed for making robust conclusions about microbial resistance and resilience (Garnier et al., 2017). Focusing on just a single aspect of community stability may hence be misleading, since a stressor can affect multiple stability components differently (Baert et al., 2016; Pennekamp et al., 2018).

## CONFLICT OF INTEREST

The authors declare no competing financial interests.

## AUTHOR CONTRIBUTION

**Marcel Suleiman:** Conceptualization (equal); Data curation (equal); Formal analysis (equal); Funding acquisition (equal); Validation (equal); Visualization (equal); Writing – original draft (equal); Writing – review & editing (equal). **Frank Pennekamp:** Data curation (equal); Formal analysis (equal); Investigation (equal); Validation (equal); Visualization (equal); Writing – original draft (equal); Writing – review & editing (equal). **Yves Choffat:** Data curation (equal); Investigation (equal); Methodology (equal); Software (equal); Writing – original draft (equal); Writing – review & editing (equal). **Owen L. Petchey:** Conceptualization (equal); Data curation (equal); Formal analysis (equal); Funding acquisition (equal); Project administration (equal); Supervision (equal); Validation (equal); Visualization (equal); Writing – original draft (equal); Writing – review & editing (equal).

## Supporting information

Appendix S1

## Data Availability

All data used in this study are openly available. All data and detailed scripts are available on zenodo (https://doi.org/10.5281/zenodo.5195092) and sequencing raw reads are available on NCBI (PRJNA731625).
