# Supplementary material for: Contrasting resistance and resilience to light variation of the coupled oxic and anoxic components of an experimental microbial ecosystem
Source: Ecol Evol. 2022 Apr 6;12(4):e8793. doi: 10.1002/ece3.8793 (PMC8986512; doi:10.1002/ece3.8793)
Supplement: Supplementary file 1 — Appendix S1 [file ECE3-12-e8793-s001.docx]

Appendix

**Appendix Figure 1** **Macroscopic appearance and experimental setup of the micro-ecosystems.**

(a) Control micro-ecosystems on day 15 and day 35. Disturbed micro-ecosystems on day 15 (stressor sample) and day 35 (long term recovery sample). (b) Technical setup (exemplary cutout) of the micro-ecosystems and the automatic oxygen-measurements at the top and bottom sensors. Oxygen was measured in 5 min intervals. Samples were taken at day 8 (prior stressor sample), day 15 (stressor sample), day 19 (short-term recovery sample) and day 35 (long-term recovery sample) at height of the top and bottom sensor, respectively.

**Appendix Figure 2 Dynamics and response of oxygen in the micro-ecosystems`bottom layers.** Oxygen concentration was recorded every 5 minutes. **(a)** Hourly mean of the oxygen concentration of the bottom sensors of the eight micro-ecosystems. Black lines represent controls, red lines represent columns incubated in darkness from day 8-15 (blue area). A recording error caused the missing oxygen data from day 23-27.

**Appendix Figure 3 NMDS analysis of the upper and lower communities of the prior-stressor sample (day 8), stressor-sample (day 15), short-term recovery sample (day 19) and long-term recovery sample (day 35).** Stress is 0.16.

**Appendix Figure 4 Relationship between the amplitude of daily fluctuations in oxygen concentration and Shannon diversity of upper layer communities without the high amplitude-low diversity replicate** in the long-term recovery sample (day 35). Black symbols represent controls, red symbols represent treated columns. Gray ribbons show the 95% confidence intervals. Pearson's product-moment correlation: p-value=0.047, correlation coefficients = -0.76.
